# Psychological Distress Among Women With Breast Cancer in Saudi Arabia: A Phenomenological Study

**DOI:** 10.7759/cureus.69701

**Published:** 2024-09-19

**Authors:** Yara A Alghamdi, Omar Alsharqi, Ahmad Ismail

**Affiliations:** 1 Radiation Oncology, Princess Noorah Oncology Center, King Abdulaziz Medical City, Jeddah, SAU; 2 Health Management, Fakeeh College for Medical Sciences, Jeddah, SAU; 3 Nursing, Fakeeh College for Medical Sciences, Jeddah, SAU

**Keywords:** breast cancer, coping strategies, psychological distress, saudi arabia, social support

## Abstract

Background: Breast cancer has a negative impact on the psychological status of women.. In Saudi Arabia, there is limited research exploring the experience of women with breast cancer regarding the psychological influence of breast cancer.

Objective: To explore the experience of women with breast cancer regarding the psychological distress of breast cancer in Saudi Arabia.

Methods: A descriptive phenomenological study was used. Semi-structured interviews were utilized to gather information from a purposive sample of 11 patients with breast cancer who were receiving treatment at the Princess Noorah Oncology Center (PNOC) at King Abdulaziz Medical City in Jeddah, Saudi Arabia. The study employed thematic analysis to discern significant themes concerning psychological stress.

Results: The study revealed that patients' psychological distress was a significant factor influencing their daily lives. This study also revealed four themes: psychological distress from the diagnosis and treatment side effects of breast cancer, daily and work-related activities reduction, the importance of social support, and the coping strategies employed by women with cancer.

Conclusion: Breast cancer leads to significant psychological distress. Interventional programs are needed to address the psychological distress that women with cancer experience and provide the necessary support for them to employ positive coping strategies.

## Introduction

Breast cancer is the most commonly diagnosed cancer among women and the second leading cause of cancer-related deaths in this demographic [1,2]. The diagnosis of breast cancer introduces significant distress for patients, who must navigate a series of challenging decisions and circumstances. From accepting the diagnosis and undergoing treatment to understanding the prognosis and managing potential side effects or relapse, the entire process can be highly stressful and lead to psychological instability and distress [1]. Many breast cancer patients experience a range of psychological symptoms throughout their treatment journey, including distress, anxiety, depression, cognitive difficulties, and issues related to body image and sexual function [3].

Distress is a broad term encompassing a range of emotions associated with symptoms of depression, anxiety, and adjustment disorders [4]. Women with breast cancer often experience elevated levels of worry, persistent fatigue, and suicidal thoughts [5,6]. Psychological distress is frequently observed in breast cancer patients [7,8] and can result in decreased self-esteem and overall well-being [9].

Although psychological distress significantly impacts the daily functioning of breast cancer patients, it is often inadequately addressed [10]. Distress levels among these patients can fluctuate at different stages of their illness [11]. Some patients may experience distress at the time of diagnosis, while others may feel increased distress after treatment due to the end of regular interactions with healthcare providers and/or side effects from treatment [1,11]. Sociodemographic factors such as younger age, living with a partner and children or only with children, and having a paid job are associated with higher levels of enduring distress. Clinically, distress is more likely to persist in patients who have undergone mastectomy, chemotherapy, or radiation, with chemotherapy showing a stronger correlation with long-term distress compared to radiation alone [1,11]. Additionally, having received psychosocial support prior to diagnosis and having two or more comorbid conditions are also strong predictors of prolonged distress [1].

The diagnosis of breast cancer significantly impacts patients' psychological and mental well-being. Among cancer patients overall, those with breast cancer have the third highest prevalence of depression, following pancreatic and head and neck cancers [1,12]. Depression rates in breast cancer patients range from 10% to 30% [12]. This condition adversely affects women’s treatment adherence, quality of life, self-care, and can reduce immune function and survival rates [13,14].

In Saudi Arabia, breast cancer is the most prevalent cancer among women. More than 50% of breast cancer cases in Saudi Arabia are diagnosed at advanced stages, compared to 20% in developed countries [15]. This results in higher mortality rates, reduced chances of a cure, and increased treatment costs [15]. Many women fear a cancer diagnosis and thus avoid screenings. Additionally, inadequate medical services and lack of awareness contribute to women not seeking timely medical evaluations and, consequently, not receiving necessary treatment [16].

In Jeddah City, approximately half of cancer patients overestimate their risk of negative outcomes, which increases their levels of distress [17]. Many women with breast cancer experience strong psychological reactions, such as feelings of intense threat, anger, and fear related to their treatment. These distress-related symptoms are associated with increased disability and a lower quality of life. Additionally, breast cancer patients may suffer from various depressive disorders, including major depressive disorder, adjustment disorder, and dysthymia. Many women also experience mood swings and prolonged periods of depression [18]. There is limited research in Saudi Arabia on how breast cancer affects women psychologically. Thus, this study aimed to explore the psychological impact of breast cancer on women in Saudi Arabia. Exploring psychological distress in individuals with breast cancer is a vital area of research with important implications for clinical practice and patient care. By pinpointing the gaps in current support and treatment resources, this research can guide the creation of targeted interventions. These interventions aim to reduce psychological distress and improve treatment outcomes and overall quality of life for those affected by breast cancer.

## Materials and methods

Design

This study utilized a qualitative, descriptive phenomenological research design in order to explore the experience of women with breast cancer regarding the psychological impact of breast cancer in Saudi Arabia.

Setting

Data were gathered from women with breast cancer at the Princess Noorah Oncology Center (PNOC) at King Abdulaziz Medical City in Jeddah, Saudi Arabia. This facility was chosen for the study due to its comprehensive breast cancer treatment program and diverse patient demographics. King Abdulaziz Medical City is a major tertiary care hospital in Jeddah, the largest urban center in the Makkah province and the second largest city in Saudi Arabia. PNOC is the leading cancer center in Western Saudi Arabia, catering to the Western, Northern, and Southern regions of the country. It includes various departments such as Adult Medical Oncology, Adult Hematology, Bone Marrow Transplantation (BMT), Pediatric Hematology/Oncology, Radiation Oncology, Palliative Care, Gynecology Oncology, and the Oncology Data & Research Unit. In 2020, PNOC handled 88,151 outpatient visits and admitted 4,361 patients across its oncology wards, which encompass chemotherapy, radiotherapy, day case units, and clinics. The center has a total capacity of 142 beds, including an 88-bed Adult Oncology Inpatient Department, a 22-bed Bone Marrow Transplant Unit, and a 32-bed Pediatric Hematology-Oncology Unit [19,20].

Sampling technique and sample size

This study involved recruiting a group of women diagnosed with breast cancer. Over a two-week period, face-to-face interviews were carried out with 11 patients. Data collection continued until no new themes emerged, indicating that data saturation had been achieved. The patients were selected using a purposive sampling method. Inclusion criteria included the following: 1) female patients above the age of 18 years; 2) diagnosed with breast cancer at stage two, three, or four; 3) started treatment for breast cancer; and 4) could communicate verbally. Women were excluded if they had other comorbidity than breast cancer.

Data Collection Procedure

After receiving the ethical approval for this study, the researchers contacted the nursing coordinator at PNOC. They explained the study’s objectives, benefits, and procedures. The nursing coordinator assisted with data collection by identifying potential participants and providing a location for the interviews. Formal written consent was obtained from participants who agreed to take part. The interviews were conducted in person using a semi-structured format, which allowed participants to discuss their experiences and perspectives in depth while also ensuring consistency with guided questions. Semi-structured questions enabled participants to explore specific topics with full freedom of response, resulting in detailed answers and unique insights. Data collection took place in December 2023, with interviews digitally recorded and transcribed verbatim.

Rigor

To ensure the trustworthiness and truthfulness of the findings, this study adhered to the four criteria established by Lincoln and Guba to maintain rigor [21]. The first criterion, credibility, was achieved by accurately and truthfully describing the breast cancer women experience regarding their psychological distress. This involved prolonged engagement and persistent observation to fully understand the context and minimize potential distortions in the data. Specific actions include 1) spending sufficient time with the participants to understand their situation and build trust and rapport; 2) engaging in peer debriefing through meetings and discussions with an expert qualitative researcher to allow for questions and critique; and 3) performing member checking by regularly verifying data and interpretations with the participants and sharing conclusions with them to ensure accuracy and provide opportunities for additional input.

The second criterion, transferability, was achieved by providing a detailed description of the participants’ experience. Recruitment continued until data saturation was reached, ensuring completeness and replicability. The primary investigator transcribed the digitally taped data, which was then reviewed by a professional researcher. Consistency in the data collection method across the 11 interviews was achieved using a semi-structured interview with prepared questions to guide the interview and allow for new information. Dependability, the third criterion, was maintained through several actions: employing a code-recode procedure where the investigator waited at least two weeks after initial coding to re-evaluate and recode the same data, ensuring consistency in findings. A PhD professor reviewed some transcribed materials to validate the themes, and any new themes identified were acknowledged and considered. Additionally, participants were asked to evaluate the extent to which the findings represented their interpretations and views. The fourth criterion, confirmability, was ensured by 1) maintaining self-awareness of the role of the researcher as the primary instrument of the study and 2) documenting additional perceptions and recollections immediately after each interview in a private room. 

Data Analysis

The semi-structured interviews were digitally recorded and transcribed verbatim. Thematic analysis, a technique for identifying recurring patterns in qualitative data, was employed to examine the data [22]. Words, phrases, and statements reflecting the experiences of women with breast cancer were identified and highlighted. These highlights were then used to develop themes and subthemes that represented their experiences. After a thorough review of all transcripts, the data were coded according to relevance. These codes were subsequently refined and organized into potential themes. An expert in qualitative research, a PhD professor, reviewed some of the transcriptions, coding, and themes, and any new themes or descriptors suggested were evaluated. The data and interpretations were shared with participants to confirm that their perspectives were accurately represented. Finally, the themes were defined and named, and a narrative structure with detailed descriptions was constructed.

Ethics

Ethical approval for the study was granted by King Abdulaziz International Medical Research Center (ro/om/2023/r0/257). Each patient received a brief explanation regarding the study’s purpose, benefits, potential risks, and methods. Written consent was obtained from all participants, including their approval for digital recording, by having them sign a form before the interview began. Participants were assured that their names would not be disclosed and that they would be identified only by a number in the study. Data were reported using numbers or codes to ensure anonymity. Participants were also informed of their right to withdraw from the study at any time and that their data would remain confidential. Participation was entirely voluntary, and participants were guaranteed that their data would not be shared with any institutions.

## Results

11 women with breast cancer participated in this study. Their age ranges from 30 to 65 years. The majority of them were unemployed (73%). Nearly half were married (55%) and held university degrees (45%) (Table 1).

**Table 1:** Participants’ demographics

Number	Age (in Years)	Marital Status	Education Level	Employment Status
1	65	Widowed	Intermediate	Unemployed
2	47	Widow	Diploma	Administrative
3	41	Married	Secondary	Unemployed
4	59	Separated	University	Own business
5	50	Married	University	Unemployed
6	50	Married	Institute	Unemployed
7	44	Widow	Diploma	Government sector
8	37	Married	University	Unemployed
9	-	Married	High school	Unemployed
10	30	Single	University	Unemployed
11	52	Married	University	Unemployed

Four themes emerged from the data analysis regarding the psychological distress related to breast cancer in women in Saudi Arabia. The first theme was that breast cancer diagnosis and treatment side effects can lead to psychological distress. The second theme was that breast cancer can reduce daily and work-related activities. The third theme was that social support mitigates the psychological distress in breast cancer patients. The fourth theme was that breast cancer women employ a variety of coping strategies.

The first theme related to the psychological distress of breast cancer diagnosis and treatment side effects. Breast cancer diagnosis led to many psychological reactions. Feeling shocked was the most common reaction experienced by half of the group (Participants 1, 2, 5, 9, and 10). Participant 5 shared, "I was in a state of shock, crying non-stop and constantly thinking about my kids." Participant 10 said, "It was the most unexpected news I had ever received, and it was the biggest shock of my life." Sadness was the second psychological distress felt by three participants (3, 7, and 8). Participant 3 said, "I was really sad and scared; however, I accepted everything as the will of Allah." Participant 8 said, "Perhaps the fear and sadness outweigh the negative effects of the medication." Two felt uncertain (Participants 6 and 7). Participant 6 said, “I was so afraid of what might come next that all I could think about was the potential courses of action and their results." Participant 7 shared, "The moment I felt the lump, I knew something was wrong; it was a sign that things were going to change” (Table 2).

**Table 2:** Psychological reactions to breast cancer diagnosis among women with breast cancer in Saudi Arabia

Participant Number	Initial Reactions to Diagnosis
1, 2, 5, 9, 10	Shock
3, 7, 8	Sadness
6, 7	Uncertain

Breast cancer's side effects also led to psychological distress in patients. They reported many side effects of breast cancer treatment that affected their physical and psychological health. These included exhaustion, fatigue, lethargy, hair loss, pain, and stress. Participant 1 said, "I found the treatments to be quite difficult. I felt the effects of the stress more strongly than I had anticipated since my body was not able to handle it as well as it once could." Participant 3 stated, “The fatigue was overwhelming. I could barely get out of bed some days." Participant 4 said, "The worst thing that happened to me was losing my hair; I had a really hard time dealing with it." Participant 6 shared, "My bones are greatly impacted by the side effects of the cancer treatment, so I was in pain all over” (Table 3).

**Table 3:** Side effects of breast cancer treatment among women with breast cancer in Saudi Arabia

Participant Number	Treatment Side Effects
1	Body exhaustion
2	Fatigue and lethargy
3	Fatigue and exhaustion
4	Fatigue and hair loss
5	Not specified
6	Lethargy and bone pain
7	Fatigue and exhaustion
10	Weight gain and tiredness

The second theme was related to the impact of breast cancer on daily and work-related activities. Daily and work-related activities reduced as a result of breast cancer. Participant 4 shared, "I made the decision to take an early retirement and concentrate on my health because I was unable to work and receive treatment." Participant 7 said, "I am exhausted and unable to perform the tasks I used to perform." Participant 6 shared, “Even though there are moments when I feel so lazy that I cannot work at all, I push myself to complete my everyday errands" (Table 4). 

**Table 4:** The impact of breast cancer on daily activities among women with breast cancer in Saudi Arabia

Participant Number	Impact of Breast Cancer on Daily Activities and Work
1	Greatly affected
2, 4, 10, 11	Affected
3	Significant impact
6	Sometimes lazy
7	Unable to engage in activities
5, 8, 9	Not specified

The third theme was related to the impact of social support on the psychological status of women with breast cancer. The more social support women with breast cancer received, the less psychological distress they experienced. Participant 1 said, "My son is the only one who is aware of my illness, and I feel very comfortable keeping it that way." Participant 10 said, "I had the opportunity to receive the greatest care in another country, but I was unable to miss the support of my friends and family, who were essential to my wellness journey" (Table 5).

**Table 5:** The impact of social support on the psychological status of women with breast cancer in Saudi Arabia

Participant Number	Social Support
1	Limited support mainly from her son, highlighting minimal social interaction.
2	Family and friends played a crucial role, considered instrumental after losing her faith.
3	Predominantly supported by her family, including her mother and brothers.
4	Received support, described as supportive without specifics.
5	The level of support is not specified in the narrative.
6	Described as having supportive family and healthcare providers.
7	Family support described as crucial, no mention of external or community support.
8	Supported by her family, particularly her husband and some friends.
9	Extensive social support network, though specifics are not detailed.
10	Significant social support from friends and family.
11	Strong support from her son who took significant steps to provide care, and from other family and friends.

The fourth theme was related to the coping strategies employed by women with breast cancer. They employed many strategies to cope with breast cancer. These included practicing religious rituals, physical activities, social media, and arts and crafts. Participant 3 said, "I read the Qur'an and do my religious duties as a way to cope with that." Participant 5 shared, "I have no choice but to accept fate and destiny and read the Qur'an." Participant 1 said, "I do not do anything besides watch social media because I am constantly feeling stiff and tired." Participant 10 said, "I walk and go to the gym to ease my psychological distress." Participant 4 said, "When I work with decoupage, I forget about my illness and feel happier and more energized" (Table 6). 

**Table 6:** Coping strategies employed by women with breast cancer in Saudi Arabia

Participant Number	Coping Strategy
1	Spiritual and digital engagements, including prayer and social media interaction.
2	Spirituality and personal practices such as prayer, reading the Qur'an, and physical activities like walking and meditation.
3	Predominantly spiritual, relying on the Qur'an and religious duties.
4	Spirituality (prayer), engaging in hobbies like decoupage.
5	Acceptance of fate and destiny, regular exercise.
6	The specific strategies are not mentioned, but support from family and healthcare providers is highlighted.
7	Spiritual practices, focusing on prayer and the Qur'an.
8	Deeply rooted in spiritual practices and surrendering to God, avoidance of disease-related information.
9	Not specified but reports a general resilience and minimal psychological stress.
10	Engaging in gym activities and athletics.
11	Spiritual practices include maintaining connections with loved ones, and possibly engaging in informal support through family and friends.

## Discussion

The results of this study indicate that breast cancer diagnosis and treatment side effects can lead to psychological distress. Breast cancer may lead to reduced daily and work-related activities. Social support eases the psychological distress in women with breast cancer. Women with breast cancer use several coping strategies to adapt to breast cancer, including religious practices, physical activities, social media platforms, and decoupage.

Research from various countries demonstrates that breast cancer frequently results in psychological distress [8,23,24]. Common forms of psychological distress among these patients include symptoms of depression and anxiety. A recent study from Oman published in 2023 found that over 20% of women with breast cancer exhibited signs of depression and anxiety [23]. The likelihood of depression rises in women newly diagnosed with breast cancer [24]. Similarly, a Greek study reported that about one-third of women with breast cancer experienced depression and anxiety symptoms [8]. A study from Sweden highlighted that psychological distress impacts the health-related quality of life for women with breast cancer, affecting aspects such as body image, future outlook, side effects of systemic therapy, and symptoms related to the breast, arm, and hair loss [24]. Additionally, research from the United States found that women with breast cancer often feel shocked by their diagnosis, associating it with a sense of impending hopelessness and death [25]. Our qualitative study reveals that women with breast cancer experience a range of distressing emotions, including shock, sadness, fear, uncertainty, exhaustion, fatigue, lethargy, pain, and stress, with anxiety and depressive symptoms being predominant. It also indicates that psychological distress can arise at the time of diagnosis or treatment, particularly due to treatment side effects. As the disease progresses, requiring more aggressive treatments, patients may face unpleasant side effects like hair loss, disfigurement, decreased libido, and nausea, all of which can negatively impact body image and increase psychological distress [23].

Several research studies from different countries indicate that breast cancer can impact the quality of life of women [24,26-28]. Our findings align with these studies. For instance, research from Iran highlights that psychological distress impacts the quality of life for breast cancer patients, with common issues including childcare responsibilities, fear, anxiety, difficulties with bathing and dressing, family problems, fever, and nasal dryness [27]. Similarly, a study from Sweden reports that psychological distress affects women’s health-related quality of life concerning body image, future outlook, side effects of systemic therapy, and symptoms related to breast, arm, and hair loss. Women undergoing chemotherapy or breast reconstruction experienced increased depressive symptoms, as these treatments negatively impacted their body image [24]. Research from Denmark found that breast cancer severely diminishes physical quality of life and activity levels [26]. Additionally, studies from Canada and Iran show that cancer diagnosis and treatment lead to a significant reduction in daily activities [28]. In our study, women with breast cancer reported reduced daily and work-related activities due to the disease, which may contribute to lower quality of life and higher psychological distress.

Social support improved the experience of women with breast cancer [29-32]. Our study's findings align with previous research. An Iranian study found that social support alleviates psychological distress in women with breast cancer, with social support being negatively correlated with depression and anxiety, underscoring its importance for improving psychological well-being [32]. A more recent Iranian study highlighted a statistically significant link between social support and a sense of life meaning, suggesting that greater social support enhances life meaning for women with breast cancer [30]. Research from Taiwan indicated that social support from family members and health professionals mediates the relationship between body image distress and resilience, emphasizing the need for increased social support to improve the experiences of women with breast cancer [31]. A study from Ghana found that spouses and children provided the most support to women with breast cancer, suggesting that integrating social support into management plans is crucial for reducing psychological distress [29]. In our study, higher levels of social support, particularly from family and friends, were associated with lower levels of psychological distress among women with breast cancer.

Research shows that women with breast cancer employ coping strategies to cope with the psychological distress from breast cancer diagnosis and treatment. Our study's findings are in line with previous research. A study from Ghana identified that the most common coping strategies among women with breast cancer include religious coping, planning, acceptance, and humor. It is important to note that the choice of coping strategies is influenced by the level of social support received [29]. Another Iranian study revealed that women with breast cancer use a range of coping strategies, both positive and negative, such as isolation, fatalism, projection, avoidance, cognitive acceptance, positive thinking, seeking spiritual support, maintaining roles, and utilizing available support [33]. Research from Ethiopia found that most women with cancer employed confronting and distancing techniques to manage psychological distress. Enhanced social support was linked to more positive coping strategies, highlighting its role in alleviating distress from breast cancer [34]. A study in Saudi Arabia found a significant positive correlation between religiosity and psychological resilience in women with breast cancer [35]. In our study, women with breast cancer used religious practices, physical activities, social media, and arts and crafts as coping mechanisms to handle their psychological distress.

To our knowledge, this is the first qualitative study in Saudi Arabia that explores the psychological distress experienced by women with breast cancer. This research offers valuable insights into their psychological reactions, the effects of distress on daily life, the role of social support, and the coping strategies used by Saudi women with breast cancer. However, the study has several limitations that may impact the generalizability of the findings beyond the sample. As a qualitative study, it is based on the subjective experiences of the participants. It was conducted at a single center with a small group of women, and it did not include statistical tests to confirm the effects of social support and coping strategies. Future research could benefit from a larger-scale quantitative study across multiple locations in Saudi Arabia, coupled with statistical analysis, to better understand the impact of social support and coping mechanisms on the psychological distress of women with breast cancer.

## Conclusions

Women with breast cancer in Jeddah, Saudi Arabia, experienced psychological distress from the diagnosis and treatment side effects of breast cancer. The most common psychological reactions were feeling shocked, sad, fearful, stressed, and uncertain. Daily and work-related activities decreased as a result of breast cancer. Social support has a positive impact on the psychological distress of women with cancer. Religious practices, physical activities, social media, and arts and crafts were the coping strategies used by women with cancer to adapt to the psychological distress of breast cancer. Interventional programs are needed to address the psychological distress that women with cancer experience and provide the necessary support for them to cope with psychological distress positively. More research is needed to recruit more participants from other cities in Saudi Arabia to examine the impact of social support and coping mechanisms on the psychological distress of women with breast cancer.
